# New software for assessment and interpretation of pulmonary function tests in adults: a comparative validation study

**DOI:** 10.3389/fphys.2026.1917497

**Published:** 2026-10-12

**Authors:** Khadija Ayed, Mhamed Acheche, Juan Del Coso, Ismail Laher, Ayoub Saeidi, Anthony C. Hackney, Hassane Zouhal

**Affiliations:** 1 Faculty of Medical Sciences, UM6P Hospitals, Mohammed VI Polytechnic University, Benguerir, Morocco; 2 Institut International des Sciences du Sport (2IS), Rennes, France; 3 Faculty of Medicine of Sousse, Sousse, Tunisia; 4 Sport Sciences Research Centre, University of Rey Juan Carlos, Madrid, Spain; 5 Institute of Health and Sport Sciences, Faculty of Health Sciences, Universidad Francisco de Vitoria, Madrid, Spain; 6 Department of Anesthesiology, Pharmacology, and Therapeutics, Faculty of Medicine, University of British Columbia, Vancouver, BC, Canada; 7 Department of Physical Education and Sport Sciences, Faculty of Humanities and Social Sciences, University of Kurdistan, Sanandaj, Kurdistan, Iran; 8 Department of Exercise & Sport Science, University of North Carolina, Chapel Hill, NC, United States

**Keywords:** automated interpretation, diagnostic concordance, guideline implementation, inter-observer variability, medical software validation, pulmonary diagnostics, respiratory physiology testing

## Abstract

**Background and objectives:**

Despite standardized international guidelines, interpretation of pulmonary function tests (PFTs) remains subject to substantial inter-observer variability. Automated guideline-based decision support systems may improve the standardization and reproducibility of PFT reporting. This agreement study evaluated the concordance between the interpretation generated by a new automated interpretation software (DrPFT^©^: Doctor Pulmonary Function Test) and an expert-based American Thoracic Society/European Respiratory Society (ATS/ERS) classification of PFTs.

**Method:**

This non-interventional and agreement study included a cohort of 1,819 adult Tunisian participants who underwent complete PFT in outpatient and hospital settings in 2025 by a panel of five experienced pulmonologists (*n* = 3) and physiologists (*n* = 2) using the standardized ATS/ERS interpretative criteria. A consensus classification was established for ventilatory patterns (normal, obstructive, restrictive, or mixed). The same datasets were subsequently analyzed by the DrPFT^©^, which implements ATS/ERS interpretative algorithms in an automated manner. Diagnostic concordance between DrPFT^©^ and expert interpretation was assessed using sensitivity, specificity, positive, and negative predictive values, with ATS/ERS-based expert consensus serving as the reference standard.

**Results:**

Based on the ATS/ERS criteria, 26.0% of participants were classified as normal, 25.8% restrictive, 26.4% obstructive, 9.4% mixed, and 12.4% nonspecific ventilatory pattern. When compared with the ATS/ERS reference, pulmonologists/physiologists demonstrated high sensitivity across all classical patterns (90.7%–94.2%) and high specificity, particularly for restrictive (99.3%) and mixed (98.0%) patterns. DrPFT^©^ showed similarly robust performance when compared with the ATS/ERS reference, with sensitivities ranging from 88.6% to 93.6% and specificities ≥84.5% for all ventilatory patterns. Direct comparison revealed excellent agreement between DrPFT^©^ and expert interpretation, with sensitivity ≥96.3% and specificity ≥98.4% across all categories. Sensitivity was comparable between DrPFT^©^ and expert interpretation for restrictive, obstructive, and mixed patterns (all *p* > 0.050), whereas a small but statistically significant difference was observed for the classification of normal pulmonary function pattern participants, which was higher via expert interpretation than via DrPFT^©^ (90.7% vs. 88.6%, respectively; *p* = 0.002).

**Conclusions:**

DrPFT^©^ provides automated ATS/ERS guideline-based interpretation of ventilatory patterns using spirometry and lung volume measurements, with a level of concordance comparable to experts. Although the software also incorporates interpretation of carbon monoxide diffusing capacity (DLCO), this component was not formally validated in the present study because of insufficient data. By standardizing the application of ATS/ERS interpretative criteria, this decision support system may improve the consistency and efficiency of routine PFT reporting, particularly in settings with limited access to specialized expertise.

**Clinical trial number:**

Pan African Clinical Trials Registry (PACTR) Identifier: *PACTR202601512810753.*

## Introduction

Pulmonary function tests (PFTs) constitute a cornerstone in the clinical assessment of respiratory physiology (Wanger et al., 2005). A comprehensive evaluation generally comprises spirometry, measurements of static lung volumes, and the assessment of pulmonary diffusing capacity for carbon monoxide (DLCO). The interpretation of PFTs aims to identify physiological abnormalities and classify them into different ventilatory profiles. This process requires comparing measured values with appropriate reference standards and applying interpretation algorithms established by working groups of the American Thoracic Society (ATS) and the European Respiratory Society (ERS) (Macintyre et al., 2005; Miller et al., 2005b; Wanger et al., 2005; Stanojevic et al., 2022). The severity of functional impairment, if any, is then quantified either as a percentage of the predicted value or as the standardized distance from the predicted mean, expressed as a *z*-score. To establish a clinical interpretation, PFT results are integrated with patient demographic and clinical information, reported symptoms, and, when appropriate, complementary investigations. Despite this rigorous standardization, significant inter-observer variability in the interpretation of PFTs persists, which can lead to inconsistent diagnostic labeling, reduced reproducibility between centers, and potential implications for clinical decision-making.

The interpretation of PFTs remains a cognitively demanding task, particularly when complex multistep algorithms need to be applied in routine clinical practice. For this reason, the ATS/ERS task force proposed a simplified computer-based algorithm for the clinical interpretation of PFTs (Pellegrino et al., 2005). However, when these guideline-based recommendations were implemented in diagnostic decision support software, the accuracy of disease classification reached only 38% (Topalovic et al., 2017). The integration of patient-specific clinical characteristics into the algorithm markedly improved diagnostic accuracy to 68%, highlighting the substantial potential of automated diagnostic labeling when PFT data are combined with clinical information (Decramer et al., 2013; Topalovic et al., 2017).

These findings suggest that automated approaches can facilitate the standardized interpretation of PFT data. However, their agreement with the interpretation of PFT experts requires further evaluation under standardized conditions.

The increasing use of computerized systems has led to a proliferation of automated decision support systems in many fields, including healthcare. PFTs generate a large number of numerical parameters that can be analyzed objectively and systematically by computer. By enabling the automated and consistent application of complex interpretation rules, these systems offer promising prospects for standardizing PFT interpretation in accordance with the international ATS/ERS guidelines (Miller et al., 2005a). However, the clinical utility of automated PFT interpretation depends on its concordance with the interpretation established according to guidelines and its reproducibility when applied to clinical data.

In this context, Doctor PFT**^©^** (DrPFT**^©^**) is a software developed as a decision support informatics tool designed to assist pneumologists in the standardized interpretation of PFTs according to ATS/ERS recommendations. However, despite the growing availability of automated interpretation systems, such as DrPFT**^©^**, their real-world performance based on standardized guideline criteria and their level of diagnostic concordance with expert pneumologists have not yet been sufficiently evaluated in large clinical cohorts.

This study therefore evaluated the agreement of DrPFT^©^ with expert guideline-based interpretation of PFTs. Specifically, the classification of spirometric data into standardized functional profiles generated by DrPFT^©^ was compared with reference classification provided by pulmonologists and physiologists with extensive expertise in PFT analysis.

## Methods

### Study design

In this non-interventional validation and agreement study, we assessed the diagnostic concordance between expert guideline-based interpretation and an automated PFT interpretation system DrPFT^©^. Five experienced pulmonologists and physiologists from tertiary hospitals in Tunisia independently evaluated complete PFT datasets, including pre- and post-bronchodilator spirometry and whole-body plethysmography for lung volumes and airway resistance, together with limited clinical information (smoking history, cough, sputum production, dyspnea, and current treatment). All interpretations were performed according to standardized ATS/ERS interpretative criteria. Initial readings were conducted individually, followed by multidisciplinary consensus meetings in which discrepant classifications were reviewed to establish a final expert reference classification for each case. Ventilatory patterns were categorized as normal, obstructive, restrictive, mixed, or nonspecific according to ATS/ERS definitions (Stanojevic et al., 2022). The same anonymized datasets were then analyzed by DrPFT^©^ using an identical interpretative framework. Finally, concordance between automated and expert classifications was evaluated to determine the reliability of automated guideline implementation in routine clinical data.

All PFT datasets were anonymized before interpretation. The expert readers were blinded to the DrPFT^©^ outputs, and DrPFT^©^ processed the same anonymized datasets independently without access to the experts’ individual or consensus interpretations. Comparisons between the software and expert consensus were performed only after both interpretation processes had been completed.

### Study population

The study included a random sample of 1,819 participants prospectively collected at the outpatient clinic and hospitals of the region of Tunis and Sousse (Tunisia) in 2025 (**Figure 1**).

**Figure 1:**
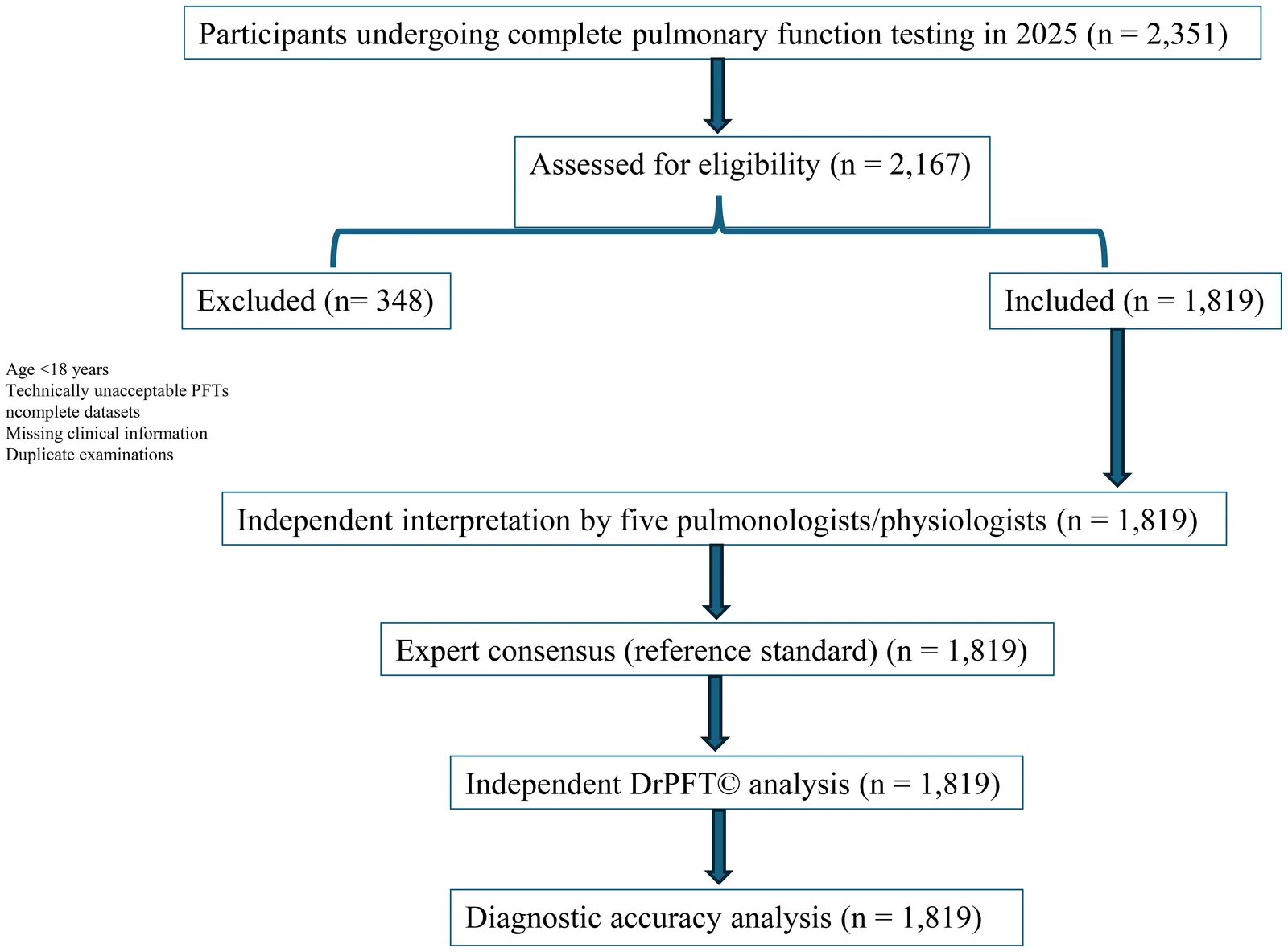
STARD flow diagram of participant selection and diagnostic accuracy study design. Flow diagram illustrating participant recruitment, eligibility assessment, exclusions, and the final analytical sample included in the diagnostic accuracy study. Adult participants undergoing complete pulmonary function testing in outpatient clinics and hospitals in Tunisia during 2025 were screened for eligibility. Participants younger than 18 years, those with technically unacceptable pulmonary function tests according to ATS/ERS quality criteria, incomplete datasets, missing clinical information, or duplicate examinations were excluded. The final study population comprised 1,819 participants. Each pulmonary function test dataset was independently interpreted by five experienced pulmonologists/physiologists, followed by expert consensus to establish the ATS/ERS-based reference standard. The same anonymized datasets were subsequently analyzed independently by DrPFT**^©^**, and diagnostic accuracy analyses were performed by comparing software classifications with the expert consensus reference standard.

Inclusion criteria comprised adults (≥18 years of age) undergoing complete pulmonary function testing with technically acceptable examinations fulfilling ATS/ERS acceptability and reproducibility criteria and complete clinical information required for interpretation. Exclusion criteria included technically unacceptable PFTs, incomplete datasets, duplicate examinations, and examinations with missing physiological variables preventing standardized ATS/ERS interpretation. No exclusions were made according to the underlying respiratory disease to reflect routine clinical practice.

All participants were Tunisian adults aged ≥18 years who underwent a complete PFT and provided relevant clinical information as part of routine clinical care. Testing was performed in the context of evaluation of respiratory symptoms, scheduled follow-up of chronic respiratory diseases, or during hospitalization for acute decompensation or exacerbation of an underlying respiratory condition. The expert reference classifications were established by a panel of five experienced pulmonologists and physiologists in Tunisia who independently reviewed complete PFT datasets together with limited clinical and anamnestic information. All classifications were performed strictly according to ATS/ERS interpretative standards. Discrepant initial readings were resolved by multidisciplinary consensus to generate a final standardized reference classification for each case within five ventilatory pattern categories: normal, obstructive, restrictive, mixed, or nonspecific. The Ethics Committee of the University Hospital Frahat Hached, Sousse, Tunisia, approved the study protocol (approval number 06112025). All included patients provided written informed consent for the use of their data. The trial was retrospectively registered (14 January 2026) in the Pan African Clinical Trials Registry (PACTR; Identifier: *PACTR202601512810753*).

### ATS/ERS reference criteria for ventilatory pattern classification

Ventilatory pattern classification was performed using a standardized, stepwise interpretative algorithm based on ATS/ERS technical standards, ensuring uniform application of predefined physiological thresholds across all participants (Stanojevic et al., 2022). A participant was classified as normal pulmonary function pattern when all spirometric and lung volume measurements were within the normal range, defined as being above the Lower Limit of Normal (LLN) for their age, sex, height, and ethnicity. Obstruction was identified when the forced expiratory volume in 1 s (FEV_1_)/forced vital capacity (FVC) ratio was below the LLN, indicating airflow limitation. Restriction is defined by a total lung capacity (TLC) below the LLN, indicating decreased lung expansion. A mixed ventilatory defect was diagnosed when both obstruction and restriction are present simultaneously within the same participant. A nonspecific ventilatory pattern was assigned when participants presented abnormal spirometric values (i.e., FEV_1_ and/or FVC below the LLN) in the absence of both airflow obstruction (normal FEV_1_/FVC) and restriction (normal TLC).

### DrPFT^©^ software

The development of software for automated reading of PFTs was performed in two versions, available in English and French. DrPFT**^©^** operates according to a structured algorithmic workflow based on ATS/ERS guidelines for PFT interpretation (Stanojevic et al., 2022). DrPFT^©^ integrates both limited clinical information and complete PFT data to support automated guideline-based interpretation. Clinical inputs include age, sex, smoking history, respiratory symptoms (cough, sputum production, and dyspnea), and ongoing treatments, while physiological inputs comprise spirometry performed before and after bronchodilator administration, static lung volume measurements, and assessment of carbon monoxide diffusing capacity (DLCO) (**Figure 2**).

**Figure 2:**
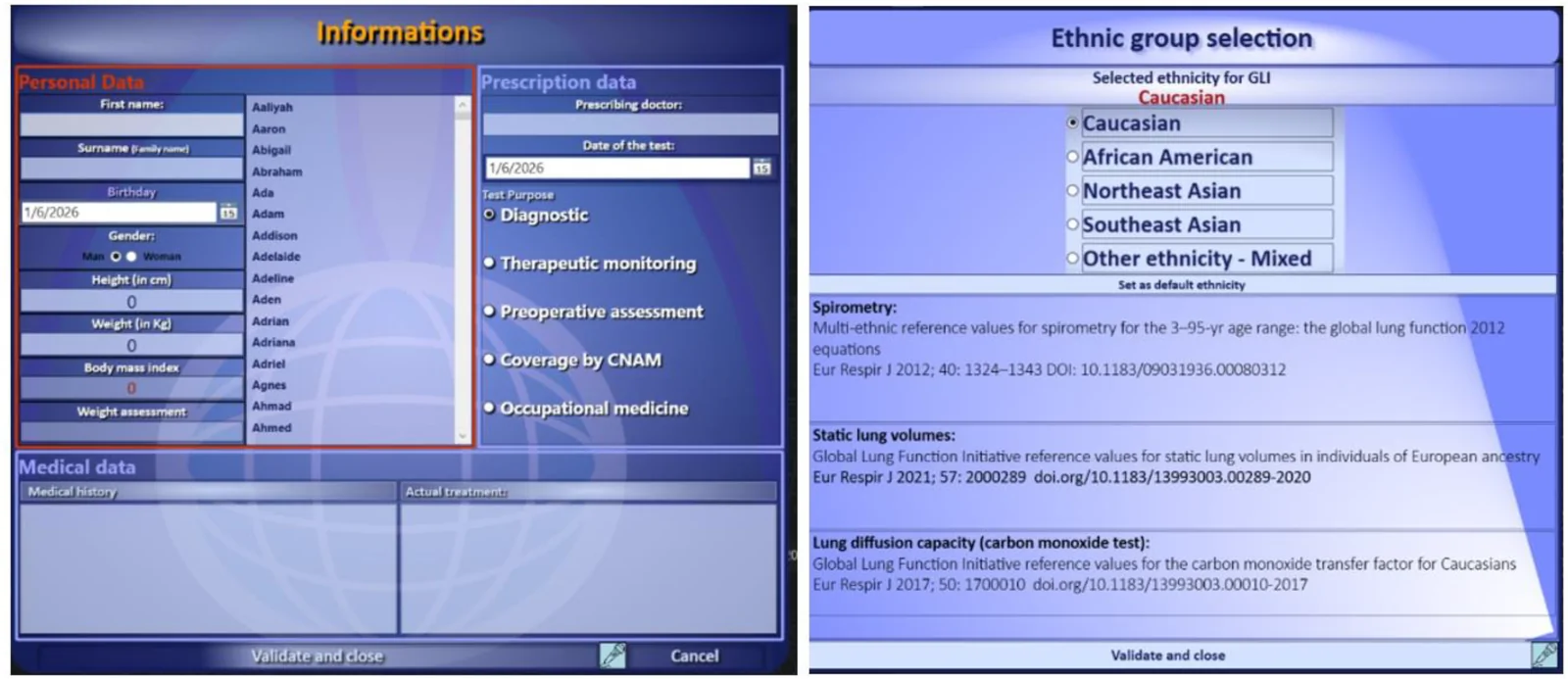
The patient’s clinical data to be entered into DrPFT^©^ software.

DrPFT^©^ was developed as a stand-alone clinical decision support application with a graphical user interface designed for routine pulmonary function laboratory use. The software incorporates Global Lung Function Initiative (GLI) reference equations for spirometry, lung volumes, and diffusing capacity to generate predicted values and *z*-scores for all measured parameters. Its internal algorithms strictly implement ATS/ERS interpretative standards, enabling automated recognition of ventilatory patterns, standardized assessment of functional impairment using *z*-scores, and algorithm-based pathophysiological orientation. DrPFT^©^ supports interpretation of spirometry with or without bronchodilator testing, static lung volumes, and diffusing capacity for carbon monoxide. For transparency, the software displays the full algorithmic decision pathway leading to each classification. Two report formats were available: a detailed report documenting all interpretative steps and a summary report providing the final classification only, allowing flexible integration into routine clinical workflows.

DrPFT^©^ analyzes the same physiological and clinical data that were provided to expert readers. Inputs included complete PFT parameters such as FVC, FEV_1_, mid- and end-expiratory flows (FEF25%–75%, FEF25%, FEF50%, and FEF75%), TLC, functional residual capacity (FRC), residual volume (RV), diffusing capacity for carbon monoxide (DLCO), and transfer coefficient (KCO). Clinical variables entered into the software include age, sex, height, weight, ethnicity, smoking exposure in pack-years, comorbidities, indication for testing, and current treatments (**Figures 2**, **3**). The software automatically derives key indices, including the FEV_1_/FVC and RV/TLC ratios, and calculates body mass index (BMI). Using GLI reference equations for spirometry, lung volumes, and DLCO, DrPFT^©^ computes predicted values, lower and upper limits of normal, and *z*-scores for all measured parameters. Each variable is then compared with its reference range to determine normality or abnormality (**Figure 4**). Based on this standardized preprocessing, the software applies ATS/ERS-defined stepwise algorithms to classify ventilatory patterns as normal, obstructive, restrictive, or mixed, with the option to display the applied decision pathway to the user (**Figure 5**).

**Figure 3:**
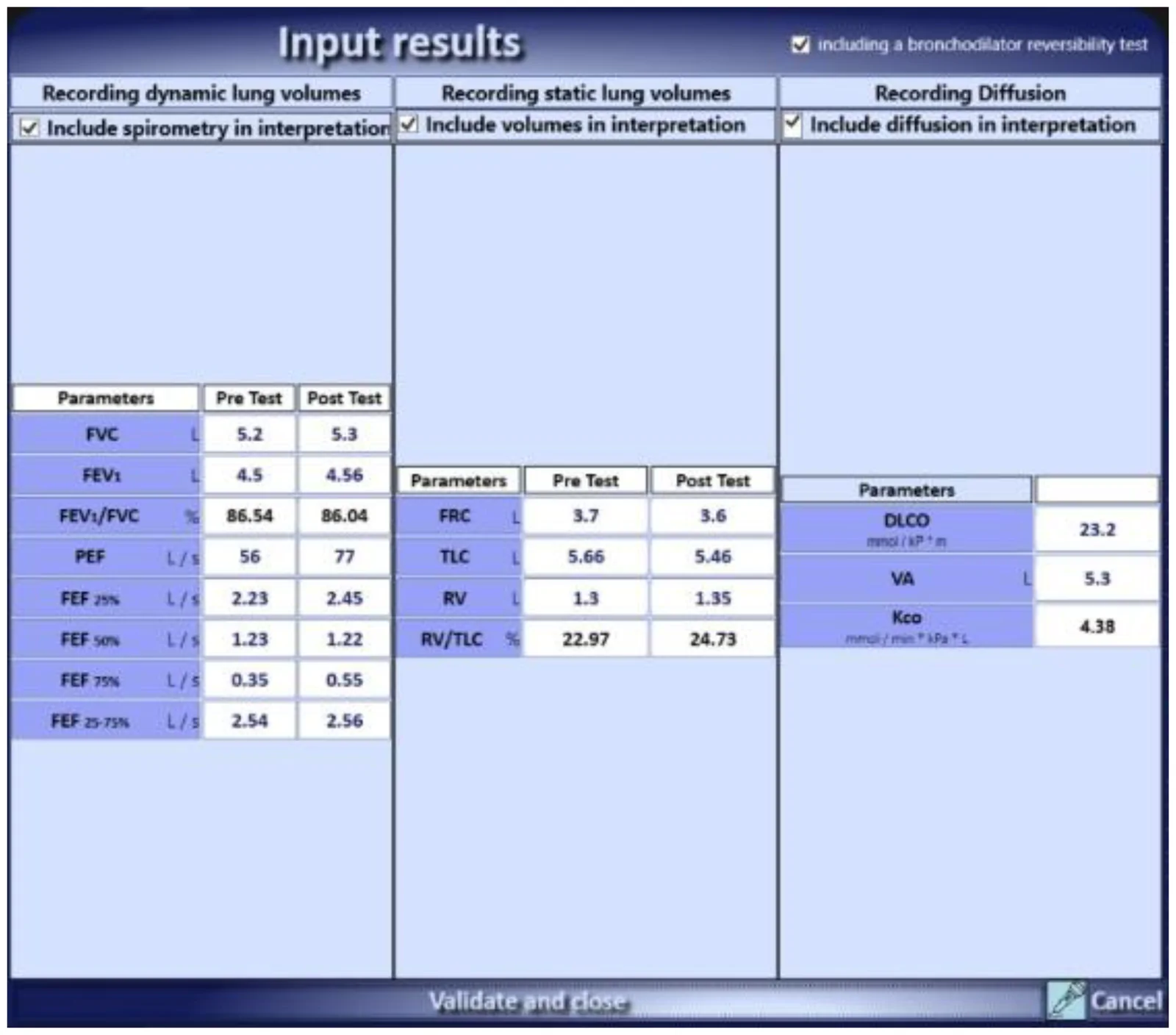
Input results (all spirometric parameters measured).

**Figure 4:**
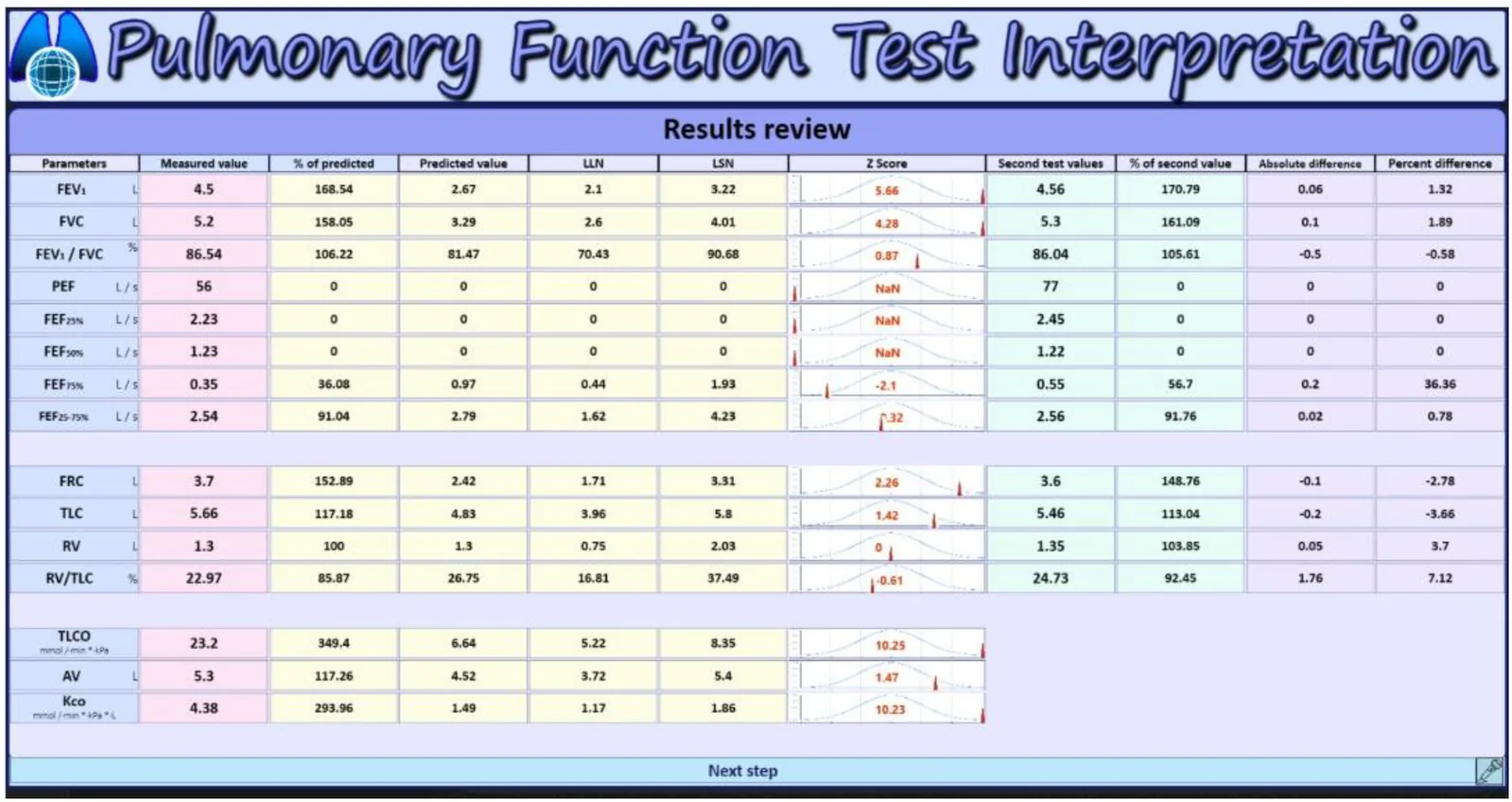
Interpretation of the DrPFT software according to the LLN and the *z*-score.

**Figure 5:**
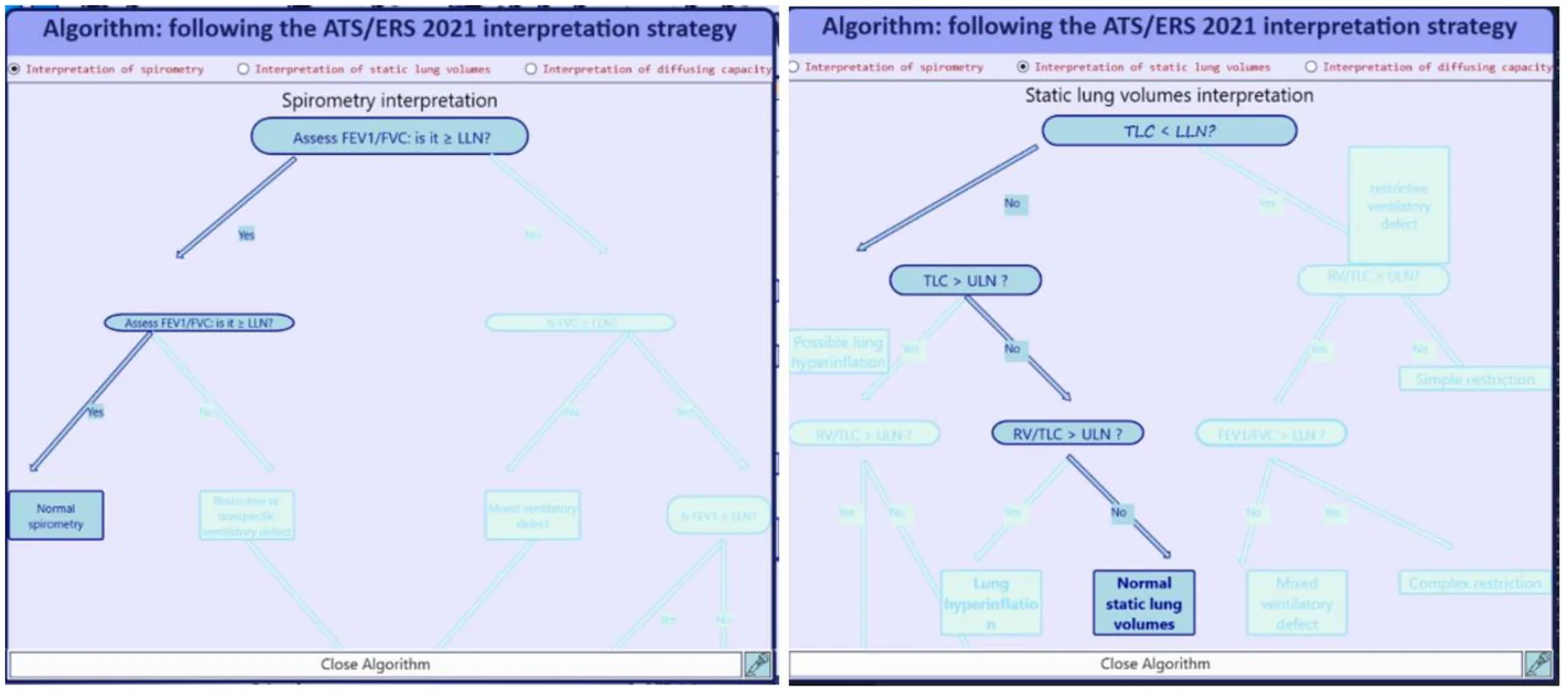
Algorithmic approach to the DrPFT^©^ software.

Following ventilatory pattern classification, DrPFT^©^ provided an algorithm-based orientation regarding the most plausible underlying respiratory mechanism and grades the severity of functional impairment using ATS/ERS-recommended *z*-score thresholds. The software then generates either a detailed interpretative report documenting each algorithmic step or a summary report presenting the final classification only (**Figure 6**). At all stages, DrPFT^©^ operates as a deterministic rule-based system that strictly implements ATS/ERS interpretative standards, ensuring consistent and reproducible guideline application.

**Figure 6:**
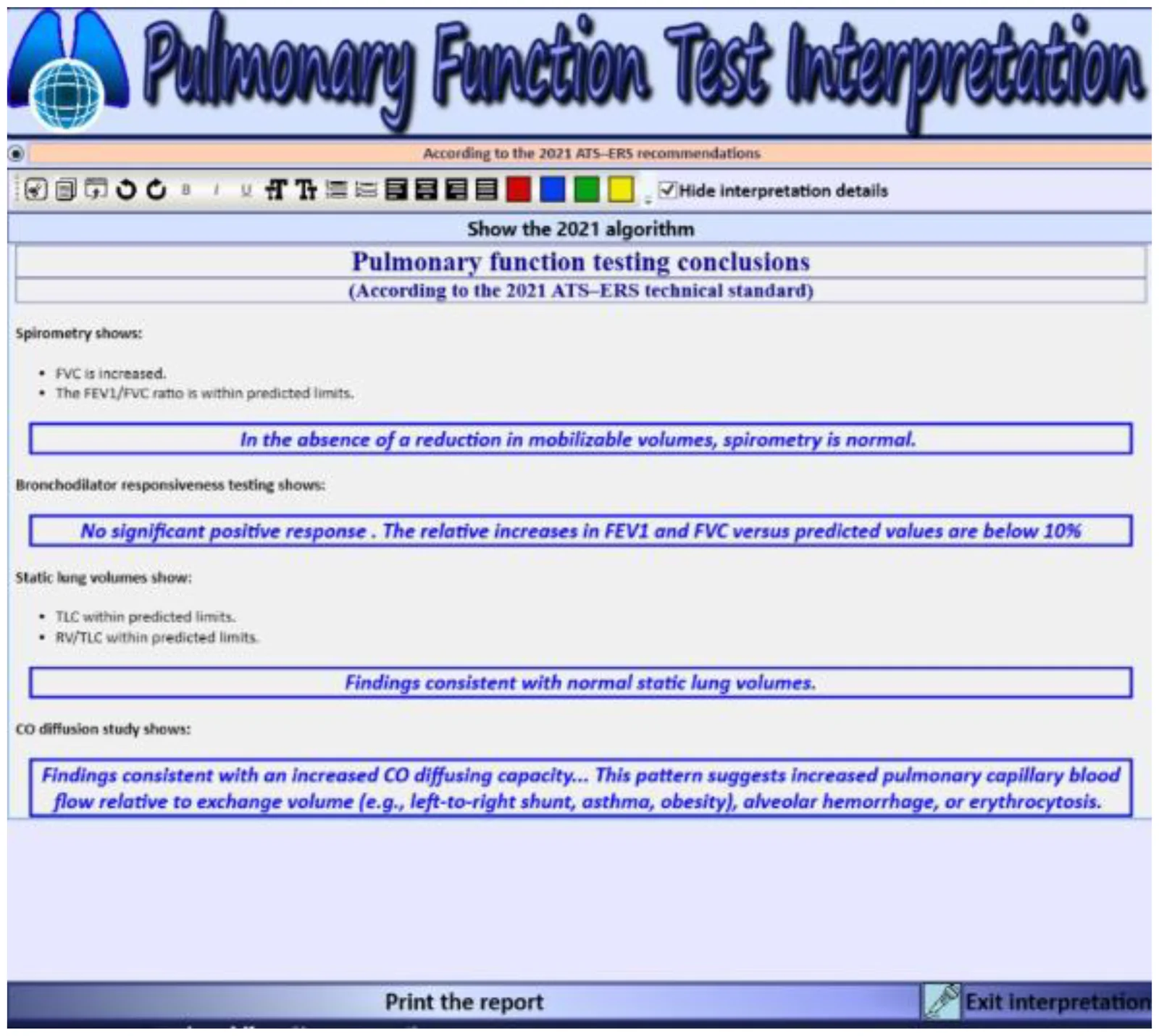
DrPFT^©^ software’s interpretation report.

The current validation focused on the software’s ability to implement ATS/ERS interpretative algorithms for ventilatory pattern classification. Although patient-specific clinical variables are incorporated into the software to provide contextual diagnostic orientation, the incremental diagnostic value of these variables beyond pulmonary function measurements alone was not specifically evaluated in the present study.

### Statistical analysis

Descriptive statistics were used to summarize the characteristics of the study sample, with continuous variables presented as mean ± standard deviation and categorical variables as counts and percentages. Ventilatory patterns (normal pulmonary function pattern, restrictive, obstructive, mixed, and nonspecific) were defined according to ATS/ERS reference criteria. To evaluate the diagnostic performance of pulmonologists/physiologists and the software DrPFT**^©^**, confusion matrices were constructed by comparing each method’s classification with the ATS/ERS reference standard (Stanojevic et al., 2022). Diagnostic performance was assessed using sensitivity, specificity, positive predictive value (PPV), and negative predictive value (NPV), calculated on a one-versus-rest basis for each ventilatory pattern. All diagnostic performance estimates are reported with corresponding 95% confidence intervals calculated using the Wilson score method. Importantly, the nonspecific ventilatory pattern was not included as a target category for either the pulmonologists/physiologists or the software DrPFT**^©^**. This decision was made because both methods are designed to classify only the four classical ventilatory patterns (normal pulmonary function pattern, obstructive, restrictive, and mixed), and do not provide an explicit nonspecific category. Consequently, participants classified as nonspecific according to ATS/ERS reference criteria were included as negative cases in the one-versus-rest analyses for the four classical patterns but were not considered true positives for any category. This approach ensured that diagnostic performance metrics reflected clinically relevant pattern recognition while remaining fully consistent with ATS/ERS interpretative guidelines (Stanojevic et al., 2022).

Because pulmonologist/physiologist and DrPFT^®^ classifications were obtained from the same participants, differences in sensitivity between the two methods were assessed using McNemar’s test for paired binary data. Absolute differences in sensitivity between DrPFT^®^ and pulmonologist/physiologist classifications were also calculated with corresponding 95% confidence intervals. Additionally, agreement between DrPFT^®^ and pulmonologist/physiologist classifications was assessed for each ventilatory pattern using pattern-specific accuracy and Cohen’s kappa (κ), with corresponding 95% confidence intervals. Accuracy was calculated as the proportion of correctly classified cases (TP + TN) among all participants. A *p*-value <0.05 was considered statistically significant. All statistical analyses were performed using R (version 4.3.2).

## Results

Demographic characteristics and pulmonary function measurements of the study sample are summarized in **Table 1**. The cohort represents a heterogeneous real-world clinical population spanning a wide adult age range and a balanced sex distribution, reflecting routine pulmonary function laboratory activity. According to ATS/ERS reference criteria, participants were classified into five mutually exclusive ventilatory patterns: 472 (26.0%) normal pulmonary function pattern, 469 (25.8%) restrictive, 481 (26.4%) obstructive, 171 (9.4%) mixed, and 226 (12.4%) nonspecific ventilatory pattern. The sample heterogeneity regarding participants’ classification provides a broad spectrum of functional profiles for the evaluation of automated and expert interpretation concordance.

**Table 1:** Descriptive characteristics of the study sample.

Variable (units)	All	Normal pulmonary function pattern	Restrictive	Obstructive	Mixed	Nonspecific
Number	1,819	472	469	481	171	226
Age (years)	52.4 ± 17.4	41.1 ± 13.8	53.1 ± 16.6	56.6 ± 17.1	64.5 ± 14.8	56.2 ± 15.7
Male/female	943/876	304/168	114/355	358/123	66/105	101/125
Height (cm)	166 ± 10	172 ± 8	161 ± 9	168 ± 9	161 ± 9	165 ± 8
Body mass (kg)	77.8 ± 16.5	81.1 ± 17.3	76.6 ± 16.8	76.8 ± 15.2	73.0 ± 16.2	79.0 ± 15.4
Body mass index (kg/m^2^)	28.2 ± 5.8	27.5 ± 5.3	29.5 ± 6.5	27.1 ± 5.3	28.3 ± 6.2	29.1 ± 5.5
FVC (L)	2.91 ± 1.09	3.91 ± 0.86	2.39 ± 0.79	2.89 ± 1.11	2.05 ± 0.70	2.56 ± 0.68
FEV_1_ (L)	2.11 ± 0.93	3.14 ± 0.69	1.93 ± 0.65	1.65 ± 0.76	1.21 ± 0.46	2.03 ± 0.57
FEV_1_/FVC (%)	72.0 ± 14.4	80.5 ± 6.3	80.5 ± 7.1	56.4 ± 10.9	58.8 ± 8.3	79.6 ± 12.9
TLC (L)	5.26 ± 1.57	6.01 ± 1.33	3.83 ± 0.91	6.24 ± 1.35	4.08 ± 0.90	5.48 ± 1.13
RV (L)	2.36 ± 1.41	2.06 ± 1.04	1.51 ± 1.34	3.36 ± 1.38	2.04 ± 0.92	2.89 ± 1.13
RV/TLC (%)	43.3 ± 17.4	33.2 ± 12.4	37.4 ± 16.8	53.1 ± 16.0	48.5 ± 18.1	51.8 ± 12.4

Data are means ± standard deviation. This categorization was based on ATS/ERS reference values.

### Comparison between pulmonologists/physiologists and ATS/ERS reference patterns

When pulmonologists/physiologists’ classifications were compared with ATS/ERS reference patterns (**Table 2**), diagnostic performance varied across ventilatory categories. Sensitivity was 90.7% for normal pulmonary function pattern individuals, 92.5% for restrictive, 91.5% for obstructive, and 94.2% for mixed patterns. Specificity was highest for restrictive (99.3%) and mixed (98.0%) patterns, and lower for normal pulmonary function pattern (83.4%) and obstructive (93.3%) categories. PPV was higher for restrictive (97.7%) and lower for normal pulmonary function pattern (65.7%), reflecting misclassification of nonspecific cases as normal. PPV for obstructive and mixed patterns was 83.0% in both cases. Overall, the pulmonologists/physiologists demonstrated strong performance across all classical ventilatory patterns, particularly for obstructive and mixed physiology.

**Table 2:** Confusion matrix with counts of all correctly and incorrectly labeled subjects per pulmonary function test, comparing the criteria of pulmonologists/physiologists with a reference pattern.

		Pulmonologists/physiologists				Total
		Normal pulmonary function pattern	Restrictive	Obstructive	Mixed	
Reference (ATS/ERS)	Normal pulmonary function pattern	428	1	43	0	472
	Restrictive	4	434	2	29	469
	Obstructive	35	2	440	4	481
	Mixed	0	7	3	161	171
	Nonspecific	184	0	42	0	226
	Total	651	444	530	194	1,819
	Sensitivity	0.907 (0.877–0.930)	0.925 (0.898–0.946)	0.915 (0.886–0.937)	0.942 (0.896–0.968)	
	Specificity	0.834 (0.814–0.853)	0.993 (0.986–0.996)	0.933 (0.918–0.945)	0.980 (0.972–0.986)	
	PPV	0.657 (0.620–0.693)	0.977 (0.959–0.988)	0.830 (0.796–0.860)	0.830 (0.771–0.876)	
	NPV	0.962 (0.950–0.972)	0.975 (0.965–0.982)	0.968 (0.957–0.976)	0.994 (0.989–0.997)	
	TP	428	434	440	161	

Sensitivity was calculated as the proportion of true cases of a given category (as per reference values) that were correctly identified as that category by the pulmonologists/physiologists. Specificity was calculated as the proportion of cases that do not belong to a given category and were correctly identified as not belonging to that category. Positive predictive value (PPV) was calculated as the proportion of predicted cases of a category that are truly in that category. Negative predictive value (NPV) was calculated as the proportion of cases predicted not to belong to a category that are truly not in that category. True positives (TP) were calculated as the number of cases correctly classified as belonging to that category. Sensitivity, specificity, PPV, NPV, and TP include 95% confidence intervals.

### Comparison between DrPFT^©^ and ATS/ERS reference patterns

Comparison of DrPFT**^©^** classifications with ATS/ERS reference patterns (**Table 3**) showed a similar but slightly attenuated performance profile. Sensitivity was 88.6% for normal pulmonary function pattern participants, 91.9% for restrictive participants, 91.1% for obstructive participants, and 93.6% for participants with mixed ventilatory patterns. Specificity remained high across categories, particularly for restrictive (98.2%) and mixed (97.8%) patterns. PPV was higher for restrictive (94.7%) and lower for normal pulmonary function pattern (66.7%). As with pulmonologists/physiologists, misclassification of nonspecific cases contributed primarily to reduced PPV in the normal pulmonary function pattern category. Overall, DrPFT**^©^** demonstrated robust performance for obstructive and restrictive patterns, with slightly lower discrimination compared with pulmonologists/physiologists.

**Table 3:** Confusion matrix with counts of all correctly and incorrectly labelled subjects per pulmonary function test, comparing the criteria of DrPFT with a reference pattern.

		DrPFT©				Total
		Normal pulmonary function pattern	Restrictive	Obstructive	Mixed	
Reference (ATS/ERS)	Normal pulmonary function pattern	418	4	50	0	472
	Restrictive	4	431	5	29	469
	Obstructive	32	4	438	7	481
	Mixed	0	8	3	160	171
	Nonspecific	173	8	44	1	226
	Total	627	455	540	197	1,819
	Sensitivity	0.886 (0.854–0.911)	0.919 (0.891–0.940)	0.911 (0.882–0.933)	0.936 (0.888–0.964)	
	Specificity	0.845 (0.825–0.863)	0.982 (0.974–0.988)	0.924 (0.908–0.937)	0.978 (0.969–0.984)	
	PPV	0.667 (0.629–0.702)	0.947 (0.923–0.964)	0.811 (0.776–0.842)	0.812 (0.752–0.861)	
	NPV	0.955 (0.941–0.965)	0.972 (0.962–0.980)	0.966 (0.955–0.975)	0.993 (0.988–0.996)	
	TP	418	431	438	160	

Sensitivity was calculated as the proportion of true cases of a given category (as per reference values) that were correctly identified as that category by the DrPFT© software. Specificity was calculated as the proportion of cases that do not belong to a given category and were correctly identified as not belonging to that category. Positive predictive value (PPV) was calculated as the proportion of predicted cases of a category that are truly that category. Negative predictive value (NPV) was calculated as the proportion of cases predicted not to belong to a category that are truly not in that category. True positives (TP) were calculated as the number of cases correctly classified as belonging to that category. Sensitivity, specificity, PPV, NPV, and TP include 95% confidence intervals.

### Agreement between DrPFT^©^ and pulmonologists/physiologists

Direct comparison between DrPFT**^©^** and pulmonologists/physiologists (**Table 4**) demonstrated high concordance across all four classical ventilatory patterns. Sensitivity ranged from 96.3% for normal pulmonary function to 99.3% for restrictive patterns, with specificity ranging from 98.4% to 100%. PPV ranged from 96.1% for obstructive patterns to 100% for normal pulmonary function, while NPV ranged from 98.0% to 99.9%. Accuracy ranged from 98.2% to 99.6% with an overall classification accuracy of 97.8% when taking into account all categories (**Table 5**). Agreement analysis using Cohen’s kappa also demonstrated very high concordance between DrPFT^®^ and pulmonologists/physiologists’ classifications across all ventilatory patterns with coefficients ranging from 0.958 to 0.980. These findings indicate excellent agreement between automated and expert human interpretation.

**Table 4:** Confusion matrix with counts of all correctly and incorrectly labeled subjects per pulmonary function test, comparing the criteria of DrPFT© and taking the criteria of pulmonologists/physiologists as a reference pattern.

		DrPFT©				Total
		Normal pulmonary function pattern	Restrictive	Obstructive	Mixed	
Reference (pulmonologists/physiologists)	Normal pulmonary function pattern	627	7	17	0	651
	Restrictive	0	441	3	0	444
	Obstructive	0	6	519	5	530
	Mixed	0	1	1	192	194
	Total	627	455	540	197	1819
	Sensitivity	0.963 (0.946–0.975)	0.993 (0.980–0.998)	0.979 (0.963–0.988)	0.990 (0.963–0.997)	
	Specificity	1.000 (0.997–1.000)	0.990 (0.983–0.994)	0.984 (0.975–0.989)	0.997 (0.993–0.999)	
	PPV	1.000 (0.994–1.000)	0.969 (0.949–0.982)	0.961 (0.941–0.974)	0.975 (0.942–0.989)	
	NPV	0.980 (0.970–0.986)	0.998 (0.994–0.999)	0.991 (0.985–0.995)	0.999 (0.996–1.000)	
	TP	627	441	519	192	

Sensitivity was calculated as the proportion of true cases of a given category (as per reference values) that were correctly identified as that category by the DrPFT© software in comparison to the reference criteria, which, in this case, is the pulmonologist/physiologist. Specificity was calculated as the proportion of cases that do not belong to a given category and were correctly identified as not belonging to that category. Positive predictive value (PPV) was calculated as the proportion of predicted cases of a category that are truly in that category. Negative predictive value (NPV) was calculated as the proportion of cases predicted not to belong to a category that are truly not in that category. True positives (TP) was calculated as the number of cases correctly classified as belonging to that category. Sensitivity, specificity, PPV, NPV, and TP include 95% confidence intervals.

**Table 5:** Agreement between DrPFT® and pulmonologists/physiologists’ classifications for ventilatory patterns.

Pattern	Accuracy (95% CI)	Cohen’s κ (95% CI)
Normal pulmonary function	98.7 (98.0–99.1)	0.971 (0.960–0.983)
Restrictive	99.1 (98.5–99.4)	0.975 (0.963–0.987)
Obstructive	98.2 (97.5–98.8)	0.958 (0.943–0.972)
Mixed	99.6 (99.2–99.8)	0.980 (0.965–0.995)
Overall	97.8 (97.0–98.4)	0.969 (0.960–0.979)

CI, confidence interval; κ, Cohen’s kappa.

### Summary of diagnostic performance

McNemar’s tests were used to compare the sensitivity of pulmonologists/physiologists and DrPFT^®^ classifications because both methods were applied to the same participants. For normal pulmonary function patterns, sensitivity was slightly but significantly higher for pulmonologists/physiologists than for DrPFT^®^ (90.7% vs. 88.6%, respectively; *p* = 0.002). In contrast, no significant differences were observed for restrictive (92.5% vs. 91.9%; *p* = 0.250), obstructive (91.5% vs. 91.1%; *p* = 0.754), or mixed ventilatory patterns (94.2% vs. 93.6%; *p* = 1.000). **Figure 7** summarizes sensitivity, specificity, PPV, and NPV for pulmonologists/physiologists and DrPFT**^©^** relative to ATS/ERS reference standards. Both methods achieved their highest sensitivity for mixed ventilatory patterns, followed by restrictive and obstructive patterns. Specificity remained consistently high across all categories for both approaches. PPV was highest for restrictive patterns and lowest for normal pulmonary function pattern patterns, reflecting the influence of nonspecific ventilatory abnormalities. NPV remained high across all categories for both approaches, ranging from 95.5% to 99.4%, with the highest values observed for mixed ventilatory patterns.

**Figure 7:**
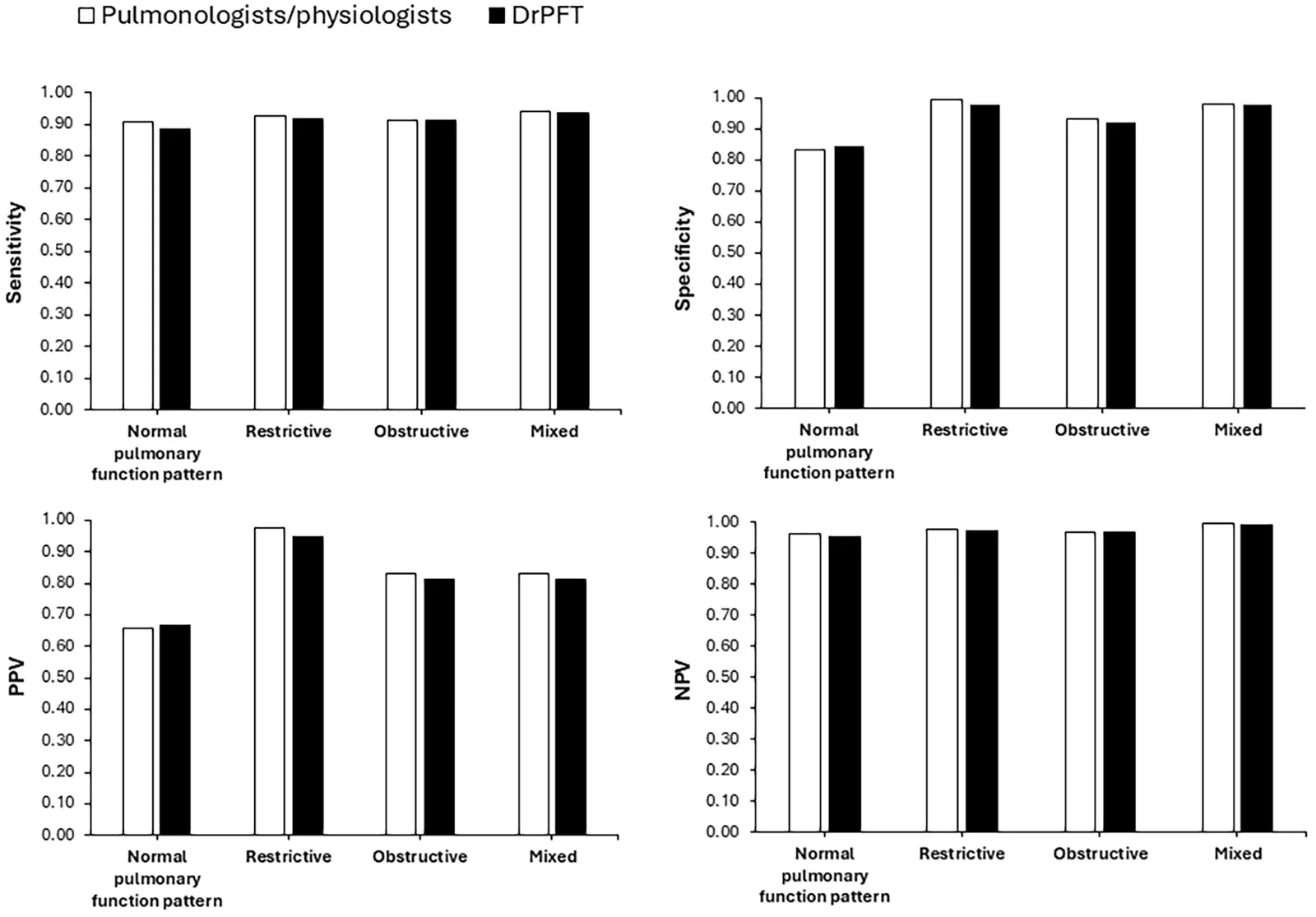
Sensitivity, specificity, positive predictive value (PPV), and negative predictive value (NPV) of pulmonologists/physiologists and the software DrPFT**^©^** in categorizing normal pulmonary function pattern individuals and those with restrictive, obstructive, or mixed ventilatory patterns based on spirometry according to ATS/ERS reference criteria.

## Discussion

Recent advances in automated computer methods have generated increasing interest in computer-assisted interpretation of PFTs. Recent studies (Waląg et al., 2023; Cowan et al., 2024; Huang et al., 2024) have explored the use of artificial intelligence and machine learning for applications such as assessing the quality of spirometry performance, detecting functional abnormalities, and predicting respiratory conditions from functional data. These approaches illustrate the potential of computer methods to facilitate the standardized analysis of pulmonary function data. In this study, the DrPFT^©^ software was developed as a clinical decision support system that implements the ATS/ERS interpretation guidelines for classifying pulmonary function profiles. Its purpose is to facilitate the standardized interpretation of PFT data, not to establish an independent clinical diagnosis or replace specialist assessment. Future developments could combine transparent recommendation-based algorithms with machine learning approaches, particularly for automated quality assessment and integration of clinical information and will need to be evaluated by appropriate external validation studies.

In this study, we demonstrated a high level of diagnostic agreement between expert guideline-based interpretation and an automated PFT interpretation system DrPFT^©^ when both were evaluated using standardized ATS/ERS criteria. Across the four classical ventilatory patterns, both approaches achieved high diagnostic performance, with the highest sensitivity observed for mixed ventilatory patterns (**Figure 7**). The paired analysis showed no significant differences in sensitivity between expert interpretation and DrPFT^®^ for restrictive, obstructive, or mixed ventilatory patterns. For normal pulmonary function pattern classifications, sensitivity was slightly higher for pulmonologists/physiologists than for DrPFT^®^ (90.7% vs. 88.6%), with this 2.1-percentage-point difference reaching statistical significance. However, the small magnitude of this difference, together with the high sensitivity observed for both approaches, suggests limited practical relevance. The inclusion of a nonspecific ventilatory pattern within the reference framework further clarified that lower PPVs for the normal pulmonary function pattern category in both methods were primarily attributable to individuals with abnormal spirometry in the absence of obstruction or restriction, rather than true misclassification of normal function (**Table 2**, **3**). Together, these results also suggest that DrPFT^©^ could be a powerful computer-based decision support tool for standardizing the interpretation of PFTs and contributing to a more consistent application of recommended interpretation criteria, while preserving the central role of expert clinical judgment in complex or ambiguous cases.

We acknowledge that the use of expert consensus as the reference standard may introduce incorporation (reference standard) bias because the consensus was generated by the same expert panel that performed the initial interpretations. To minimize this effect, each expert independently interpreted all PFTs before participating in the consensus process. The consensus was established only after completion of independent readings and served exclusively as a standardized ATS/ERS-based reference for comparison with the software. Because both the software and the reference classification relied on the same ATS/ERS interpretative framework, a degree of methodological circularity cannot be excluded. Consequently, the present study evaluates agreement between automated and expert implementation of ATS/ERS recommendations rather than comparison with an independent external gold standard.

It should also be noted that the current version of DrPFT^©^ assumes that PFTs have already satisfied ATS/ERS technical quality standards before interpretation. Consequently, the software should be considered a decision support tool for the interpretation of technically valid examinations rather than a substitute for quality control procedures. The integration of an automated ATS/ERS quality assessment module capable of evaluating acceptability and repeatability criteria before interpretation represents an important direction for future software development and should further enhance the robustness and applicability of DrPFT^©^ in routine clinical practice.

Despite the existence of interpretation guidelines, significant inter-rater variability persists in diagnostic classification and severity assessment (Miller et al., 2005a; Culver et al., 2017; Graham et al., 2017). Thus, for clinicians, assigning a precise diagnosis based solely on PFT data remains a complex task. A recent multicenter study highlighted an average accuracy of only 44.6% in the interpretation of 6,000 PFTs, with variability ranging from 39% to 51% depending on the center, and from 24% to 62% depending on the pulmonologist, reflecting frequent inter-observer disagreement (Topalovic et al., 2017). Furthermore, the aforementioned multicenter study showed no statistically significant difference based on the age or clinical experience of the examiners (45% in seniors vs. 43% in juniors), or according to the hospital (*p* = 0.44), the type of establishment (university: 44.1% vs. non-university: 45.2%), or the country (*p* = 0.26) (Topalovic et al., 2017). Another recent study showed that the identification of a restrictive pattern by pulmonologists/physiologists had a PPV of 59% and a sensitivity of 75% (Topalovic et al., 2019). This variability is partly explained by the suboptimal quality of the tests and by cognitive errors related to operator-dependent constraints, particularly fatigue when reading a very large number of PFTs (Huang et al., 2024).

In this context, the high level of concordance achieved by DrPFT^©^ compares favorably with that reported in previous studies evaluating automated systems for PFT interpretation, while differences in study design and reference standards should be taken into account (Topalovic et al., 2019; Huang et al., 2024). When evaluated against the ATS/ERS-based expert reference classifications, DrPFT^©^ demonstrated high sensitivity and specificity across all ventilatory patterns (**Table 3**; **Figure 7**). In the direct comparison with pulmonologist/physiologist interpretations, DrPFT^©^ also showed a high level of concordance, with sensitivities ranging from 96.3% to 99.3% and specificities ≥98.4% across categories (**Table 4**). PPVs ranged from 96.1% to 100% across the functional patterns evaluated, indicating a high level of concordance between DrPFT^©^ classifications and the corresponding expert-based classifications. These findings suggest that the software can provide a consistent classification of pulmonary function patterns when ATS/ERS interpretative criteria are applied through an automated, rule-based approach.

The nonspecific ventilatory pattern is recognized, within the framework of ATS/ERS interpretation, as a clinically relevant physiological pattern. In the present study, 226 participants were classified by experts as having a nonspecific pattern, whereas this category was not available as a distinct final outcome measure in the version of DrPFT^©^ being evaluated. Consequently, these cases may have contributed to predictable discrepancies and affected the performance estimates of other functional categories. Although DrPFT^©^ can identify physiological signs consistent with a nonspecific pattern during its interpretation process, explicitly considering this pattern as a distinct final classification category requires further implementation and dedicated validation. This limitation should be taken into account when interpreting the concordance results presented here and will be an important focus of future software development, enabling a more comprehensive implementation of the ATS/ERS interpretation recommendations.

The use of computerized clinical decision support systems to optimize medical practice is not a new approach. A study showed that 64% of these systems contribute significantly to improving the performance of individual clinicians (Garg et al., 2005). Currently, these computerized tools are widely integrated and used routinely across various healthcare domains (Willems et al., 1991; Filippi et al., 2003). Although several approaches to automated analysis of PFTs have been proposed and evaluated previously (Hankinson, 1981; Krumpe et al., 1982), none have yet achieved widespread clinical adoption. On the one hand, the integration of the patient’s clinical context appears essential to establish a relevant preferential diagnosis (Enright, 2006; Berry and Wise, 2009). On the other hand, the thresholds used to define functional abnormalities and to classify respiratory diseases based on PFTs remain controversial and frequently rely on arbitrary choices. This methodological heterogeneity results in significant variability in the interpretation strategies used by pulmonologists/physiologists in their daily clinical practice (Miller et al., 2011; Visentin et al., 2016).

By integrating all the complete data from respiratory function testing, the DrPFT^©^ software can identify fine, subtle, and decisive characteristics, often difficult to detect by human expertise alone, and integrate them into a high-performance discriminating diagnostic algorithm. This approach builds upon the seminal work of Topalovic et al. (2019), who demonstrated that computerized PFT interpretation software provides more accurate interpretations than conventional approaches. The authors showed 100% concordance for identifying respiratory function patterns, as well as a correct diagnosis in 82% of cases, suggesting that these tools could be powerful decision support systems to improve clinical practice. This multidimensional approach complements human capabilities for analyzing the same datasets in terms of precision and reproducibility. However, it would be unrealistic to expect 100% accuracy from software, as some respiratory pathologies are not accompanied by specific or detectable pulmonary functional abnormalities. Information processing by these artificial intelligence-based decision support systems achieves a level of performance comparable to that of a panel of experts, while offering significantly faster execution and near-total consistency when faced with identical data. The future clinical value of these tools will be fully established if they demonstrate their ability to reduce the time to a final diagnosis, limit the use of unnecessary additional tests, and, through the standardization of PFT interpretation, prevent a significant number of misdiagnoses (Topalovic et al., 2019).

Although DrPFT^©^ demonstrated high diagnostic concordance with expert interpretation, successful implementation in routine clinical practice will depend on more than diagnostic accuracy alone. Previous implementation science studies have shown that clinician acceptance, seamless integration into existing clinical workflows, interoperability with electronic health record systems, explainability of algorithmic recommendations, and adequate user training are key determinants of the successful adoption of clinical decision support systems. Future implementation studies should therefore evaluate not only the diagnostic performance of DrPFT^©^, but also its usability, clinical impact, user acceptance, and cost-effectiveness in real-world healthcare settings.

The present validation should be interpreted within the scope of the evaluated functionality. Although DrPFT^©^ was developed as a comprehensive decision support system integrating spirometry, lung volumes, and carbon monoxide diffusing capacity (DLCO), the current study formally validated only its performance for ATS/ERS-based classification of ventilatory patterns using spirometry and lung volume measurements. While the software includes an algorithm for DLCO interpretation, this component could not be formally evaluated because an adequately large and representative dataset was not available. Consequently, the current findings should not be interpreted as validation of the complete DrPFT^©^ platform, and dedicated studies focusing on DLCO interpretation are warranted before this functionality can be considered clinically validated.

Although DrPFT^©^ incorporates clinical variables to provide contextual diagnostic support, the present study was not designed to quantify their independent contribution to diagnostic performance. Future studies comparing algorithm performance with and without clinical inputs will be necessary to determine the additional diagnostic value provided by these variables.

Future multicenter studies should evaluate the diagnostic performance of DrPFT^©^ across clinically relevant subgroups, including age, sex, smoking status, disease severity, and different respiratory disorders, to further assess the robustness and generalizability of the software.

## Strengths of the study

This study has several major strengths. First, it evaluates an innovative computerized decision support system, specifically developed for the standardized interpretation of PFTs, addressing a real clinical need in the face of inter-observer variability. Second, the DrPFT^©^ algorithm relies on the multidimensional integration of all respiratory function parameters, enabling the identification of subtle characteristics often difficult to grasp through isolated human analysis. Third, the reference diagnosis was established by a panel of experts, ensuring robust and clinically relevant validation of the software’s performance. Fourth, the approach adopted allows for rapid, consistent, and reproducible interpretation of PFTs, with a level of performance comparable to that of a group of experts, while offering substantial time savings. Finally, this study adopts a clear translational perspective, proposing a tool that is immediately applicable in clinical practice and scalable, with future improvements including automatic assessment of test quality and expansion to other functional profiles defined by the ATS/ERS guidelines.

### Study limitations

This study has several limitations that should be considered when interpreting the findings.

First, the sample used in this study may not fully reflect the prevalence and diversity of respiratory function profiles encountered by pulmonologists in their daily clinical practice. In particular, the inclusion of other function profiles described in the ATS/ERS guidelines, such as respiratory muscle weakness, suboptimal effort, dysanapsia (Thompson, 2017), and the “large lung” profile, would have been desirable. These function profiles will be integrated into the diagnostic algorithms (DrPFT^©^) at a later date to improve their robustness, generalizability, and clinical relevance. Another limitation concerns the nonspecific ventilatory pattern, which was not available as a distinct final outcome category in the evaluated version of DrPFT^©^. Consequently, cases belonging to this profile could not be directly classified by the software and may have contributed to predictable discrepancies and affected the performance estimates of other functional categories. While DrPFT^©^ can identify physiological outcomes consistent with a nonspecific model during its interpretative workflow, explicitly declaring this model as a distinct final classification category requires further development and dedicated validation.

The risk of misinterpretation and misdiagnosis increases when PFTs are performed under suboptimal technical conditions (Leuppi et al., 2010). However, adequate methodological quality of the examinations is an essential prerequisite, both for human interpretation and for that provided by the decision support software. The software was unable to autonomously assess test quality. However, several studies have demonstrated the value of machine learning approaches for the objective evaluation of spirometry quality (Melia et al., 2014; Wang et al., 2022; Topole et al., 2023). The future integration of such quality indicators could contribute to strengthening the robustness and diagnostic reliability of our software.

Another limitation lies in the fact that the reference standard was based on expert consensus rather than an independent external reference standard. The experts independently interpreted PFTs according to the ATS/ERS guidelines before participating in the consensus process. Nevertheless, their classification and that of DrPFT^©^ were both based on the same ATS/ERS interpretation framework. Therefore, some methodological bias cannot be ruled out, and the current results should be interpreted as concordance between expert and automated interpretation of PFTs according to the guidelines, rather than as evidence of independent clinical diagnostic accuracy.

Although the five experts interpreted the reviews independently before reaching a consensus, formal inter-observer agreement was not assessed. Therefore, the extent of variability among the different experts cannot be fully quantified from this study. Future validation studies should include a formal assessment of inter-observer agreement using appropriate statistical measures.

Furthermore this study did not evaluate the clinical impact of DrPFT^©^ in routine practice. Specifically, it did not assess whether the software’s use alters clinical decision-making, report completion times, patient management, or clinical outcomes. Therefore, the current results do not establish the software’s clinical efficacy or impact. Prospective studies conducted in routine clinical settings will be necessary to evaluate these aspects.

Although our study included a relatively large real-world cohort, all participants were recruited from healthcare centers in Tunisia. Consequently, the generalizability of these findings to other healthcare systems, geographical regions, and ethnically diverse populations remains to be established. External validation in independent multicenter cohorts involving different patient populations, pulmonary function laboratories, and clinical settings will be essential to confirm the robustness, reproducibility, and clinical applicability of DrPFT^©^ before its widespread implementation in routine practice.

Last, although the software incorporates the interpretation of carbon monoxide diffusing capacity (DLCO), we do not yet have a sufficiently large and representative database to robustly evaluate its diagnostic performance. The specific evaluation of DrPFT^©^’s performance in interpreting DLCO will therefore be a priority for our future work.

## Conclusion

We demonstrate that DrPFT^©^ is a reliable and reproducible clinical decision support system for the automated application of ATS/ERS interpretation standards, exhibiting strong agreement with expert interpretation and enabling consistent integration of PFT data to support routine clinical reports.

The high diagnostic performance and very high Cohen’s kappa coefficients indicate that DrPFT^®^ closely reproduces expert application of ATS/ERS interpretative standards. Although expert interpretation showed a small advantage in sensitivity for normal pulmonary function pattern classifications, differences for restrictive, obstructive, and mixed patterns were not statistically significant. These findings support the use of DrPFT^®^ as a clinical decision support tool to enhance consistency in PFT reporting, while preserving the role of expert clinical judgment. Additionally, DrPFT^©^ shows potential as a new technology support system to improve clinical practice in pulmonology by optimizing both diagnostic accuracy and operational efficiency. However, it should be considered a complementary tool to clinical expertise rather than a substitute for expert assessment. Further external validation with larger populations and in independent clinical settings is needed.

The present study validates the performance of DrPFT^©^ for automated ATS/ERS-based classification of ventilatory patterns using spirometry and lung volume measurements. Although the software also incorporates interpretation of carbon monoxide diffusing capacity (DLCO), this component has not yet undergone formal validation because of insufficient data and will be evaluated in future multicenter studies. In addition, the current version of DrPFT^©^ is intended for use only with PFTs that satisfy ATS/ERS acceptability and reproducibility criteria. Automated assessment of test quality before interpretation remains an important priority for future software development and validation and will further enhance the reliability and clinical applicability of the system.

### Take-home message

Despite standardized international guidelines, interpretation of PFTs remains subject to substantial inter-observer variability.

Automated guideline-based decision support systems may improve the standardization and reproducibility of PFT reporting.

The new automated interpretation software DrPFT^©^ appears to be a reliable and reproducible clinical decision support system for automated application of ATS/ERS interpretative standards, enabling consistent integration of PFT data to support routine clinical reporting.

## Data Availability

The raw data supporting the conclusions of this article will be made available by the authors, without undue reservation.
